# A Genome-Wide Linkage Scan for Distinct Subsets of Schizophrenia Characterized by Age at Onset and Neurocognitive Deficits

**DOI:** 10.1371/journal.pone.0024103

**Published:** 2011-08-29

**Authors:** Yin-Ju Lien, Po-Chang Hsiao, Chih-Min Liu, Stephen V. Faraone, Ming T. Tsuang, Hai-Gwo Hwu, Wei J. Chen

**Affiliations:** 1 College of Public Health, Research Center for Genes and Institute of Epidemiology and Preventive Medicine, Environment and Human Health, National Taiwan University, Taipei, Taiwan; 2 Genetic Epidemiology Core Laboratory, Center of Genomic Medicine, National Taiwan University, Taipei, Taiwan; 3 Department of Public Health, College of Public Health, National Taiwan University, Taipei, Taiwan; 4 Department of Psychiatry, College of Medicine and National Taiwan University Hospital, National Taiwan University, Taipei, Taiwan; 5 Departments of Psychiatry and of Neuroscience and Physiology, SUNY Upstate Medical University, Syracuse, New York, United States of America; 6 Department of Psychiatry and the Center for Behavioral Genomics, University of California San Diego, San Diego, California, United States of America; 7 Harvard Departments of Epidemiology and Psychiatry, Harvard Institute of Psychiatric Epidemiology and Genetics, Boston, Massachusetts, United States of America; Kyushu Institute of Technology, Japan

## Abstract

**Background:**

As schizophrenia is genetically and phenotypically heterogeneous, targeting genetically informative phenotypes may help identify greater linkage signals. The aim of the study is to evaluate the genetic linkage evidence for schizophrenia in subsets of families with earlier age at onset or greater neurocognitive deficits.

**Methods:**

Patients with schizophrenia (n  =  1,207) and their first-degree relatives (n  =  1,035) from 557 families with schizophrenia were recruited from six data collection field research centers throughout Taiwan. Subjects completed a face-to-face semi-structured interview, the Continuous Performance Test (CPT), the Wisconsin Card Sorting Test, and were genotyped with 386 microsatellite markers across the genome.

**Results:**

A maximum nonparametric logarithm of odds (LOD) score of 4.17 at 2q22.1 was found in 295 families ranked by increasing age at onset, which had significant increases in the maximum LOD score compared with those obtained in initial linkage analyses using all available families. Based on this subset, a further subsetting by false alarm rate on the undegraded and degraded CPT obtained further increase in the nested subset-based LOD on 2q22.1, with a score of 7.36 in 228 families and 7.71 in 243 families, respectively.

**Conclusion:**

We found possible evidence of linkage on chromosome 2q22.1 in families of schizophrenia patients with more CPT false alarm rates nested within the families with younger age at onset. These results highlight the importance of incorporating genetically informative phenotypes in unraveling the complex genetics of schizophrenia.

## Introduction

Family and twin studies have indicated that schizophrenia has a strong genetic basis [1]. However, previous genetic linkage and association studies have not conclusively detected susceptibility loci and genetic variants for schizophrenia [2], [3]. Heterogeneity in the etiology of schizophrenia may partly account for the conflicting results. Further, the current diagnostic schemes may not capture the most heritable features of schizophrenia. To overcome these obstacles, using genetically informative phenotypes has been advocated since this may reduce heterogeneity or represent the more direct effects of underlying susceptibility genes [4]. Conceptually, a genetically informative phenotype can be a component phenotype, i.e., a symptom or dimension which is a component of the disorder itself found only in affected individuals, or an intermediate phenotype, i.e., a quantitative phenotype which is found in both affected individuals and their nonpsychotic relatives.

Cumulative evidence suggests that age at onset is one potential component phenotype for schizophrenia. Compared with schizophrenia patients with late onset, those with early age at onset have been associated with more severe cognitive impairments and poorer outcomes [5]. In addition, patients with an early onset were more likely to have relatives affected with schizophrenia [6], and a greater correlation in age at onset was found in affected monozygotic twin pairs than in dizygotic twin pairs [7]. Meanwhile, neurocognitive deficits such as sustained attention deficit and executive dysfunction are possible intermediate phenotypes for schizophrenia. Sustained attention deficits as measured on the Continuous Performance Test (CPT) [8] are present, not only in schizophrenia patients, but also in subjects with schizotypal personality disorder and in nonpsychotic relatives of schizophrenia patients [9]. Family studies have found an elevated recurrence risk ratio for CPT deficits among nonpsychotic parents or siblings [10] and a positive association between the severity of the CPT deficits and the familial loading for schizophrenia [11]. The performance deficits in schizophrenia patients are stable in clinical course and not amenable to treatment with neuroleptics [12]. Executive functioning as measured by the Wisconsin Card Sorting Test (WCST) [13] have also been found to be impaired in schizophrenia patients [14] and their first degree relatives [15], though the magnitude of familial aggregation is modest at best [16].

A number of candidate gene studies have explored the relations of certain genetic variants to schizophrenia by subgrouping patients using these genetically informative phenotypes of schizophrenia, including age at onset [17], and differential impairments on the CPT or WCST [18]. However, the subgrouping in these studies was mainly based on a predetermined cut-off, which may not result in the most homogenous subsets. An attractive alternative for exploring susceptibility loci influencing different informative phenotypes of schizophrenia is a discovery-based genome-wide scan such as ordered subset analysis (OSA), a method not requiring a priori specification of the subset [19]. Given that age at onset is only partially associated with the severity of neurocognitive deficits in schizophrenia [20], subsetting families using a combination of these phenotypes may help further reveal a more homogeneous subset of families with greater linkage signals.

This study aimed to evaluate the genetic linkage evidence for empirically derived subtypes of schizophrenia by applying ordered subset linkage analyses in a large sample of families of siblings co-affected with schizophrenia, which consisted mainly of a single ethnic group [21]. We hypothesized that using a homogeneous subtype of schizophrenia on the basis of age at onset or neurocognitive deficits can help obtain increased linkage signals as well as identify additional regions as compared with the initial genome-wide linkage scan of schizophrenia.

## Methods

### Subjects and clinical evaluation

Participants of this study included patients with schizophrenia and their first-degree relatives recruited from six data collection research center throughout the nation in the Taiwan Schizophrenia Linkage Study between 1998 and 2002. The study design, ascertainment process, and sample characteristics have been described in more detail elsewhere [22]. In brief, the large nation-wide sample of schizophrenia families were ascertained on the basis of sib-pairs co-affected with DSM-IV schizophrenia, which included only families of Han Chinese ancestry. The original linkage sample consisted of 1,207 affected individuals (57 parents and 1,150 siblings) and 1,035 unaffected individuals (764 parents and 828 siblings) from 557 schizophrenia families [23]. All subjects provided written informed consent after the study procedures were explained to them. Best-estimate final diagnoses were made by two board-certified research psychiatrists independently on the basis of all clinical information, including the Diagnostic Interview for Genetic Studies (DIGS) [24], the Family Interview for Genetic Studies (FIGS) [25], hospital records, and the interviewer's notes. The study was approved by both the US Department of Health and Human Services and the National Taiwan University Hospital's Internal Review Board of Human Studies.

The DIGS includes a psychosis section that inquires about the age at onset of the first psychotic episode that could not be attributed to medical illness, medications, or substance abuse. When the information with regard to onset of the first psychotic episode on the interview was unclear, research psychiatrists would review the medical history and determine the age at onset.

### Neurocognitive assessment

Probands and their relatives completed both the CPT and the WCST. The degraded and undegraded version of CPT -‘1-9’ procedure has been described in detail elsewhere [26]. Briefly, numbers from 0 to 9 were randomly presented for 50 msec each, at a rate of one per second. Each subject undertook two CPT -‘1-9’ sessions: the undegraded 1-9 tasks and 25% degraded 1-9 tasks. Sensitivity (d́), derived from the hit rate (probability of response to target trials) and false alarm rate (probability of response to nontarget trials), reflects an individual's ability to discriminate target stimuli from nontarget ones. In addition, the reaction time (i.e., the mean time to respond correctly) for each session was also used for the analyses.

For the WCST, we used a computerized version [27]. Subjects were required to match response cards to the four stimulus cards along one of three dimensions (color, form, or number) by pressing one of the 1 to 4 number keys on the computer keyboard. Subjects were not informed of the correct sorting principle, nor were they told when the principle would shift during the test, but they were given feedback (“Right” or “Wrong”) on the screen after each trial. The testing continued until all 128 cards were sorted. Eight performance indices as described in the WCST manual [28] were used for subsequent analyses: Total Errors, Nonperseverative Errors, Perseverative Errors, Perseverative Responses, Categories Achieved, Trials to Complete First Category, Conceptual Level Response, and Failure to Maintain Set.

The adjusted z scores of the CPT indices were derived by means of standardizing the raw scores with adjustments for sex, age, and education against a community sample of 345 individuals [26], while adjusted z scores of the WCST with adjustments for sex, age, and education derived for individual against another group of 440 healthy controls [29]. Because some adjusted z scores were extremely small (<−5) or large (>5), these scores were winsorized as −5 or 5, respectively.

### Genotyping

The original genotyping was conducted by the Center for Inherited Disease Research, with 386 markers spaced at an average of 9-cM intervals, following the center's standard genotyping procedures (http://www.cidr.jhmi.edu). A series of procedures for quality control was adopted for the genotyping, such as duplicate assays with a discordancy rate of 0.06%, and checking for non-Mendelian inheritance and pedigree inconsistencies with an overall rate of 0.39% in genotype and family errors [21]. Erroneous genotypes were removed accordingly. Marker distances were generated by using the sex-averaged Marshfield genetic map. The data included for this study were collected through NIMH's genetic initiative for schizophrenia and details about how to access these data can be found at the following website: http://zork.wustl.edu/nimh.

### Statistical analysis

#### Ordered subset linkage analyses

Based on the original sample of genome-wide linkage analysis for schizophrenia, a series of OSAs [19] were conducted using nonparametric logarithm of odds (LOD) scores by means of Kong and Cox linear model in affected pedigree members [30] derived from software Merlin [31] as input. The method ranks each family by a family-level value of a disease-related covariate of interest and identifies the contiguous subset of families that maximize the evidence for linkage.

**Figure 1:**
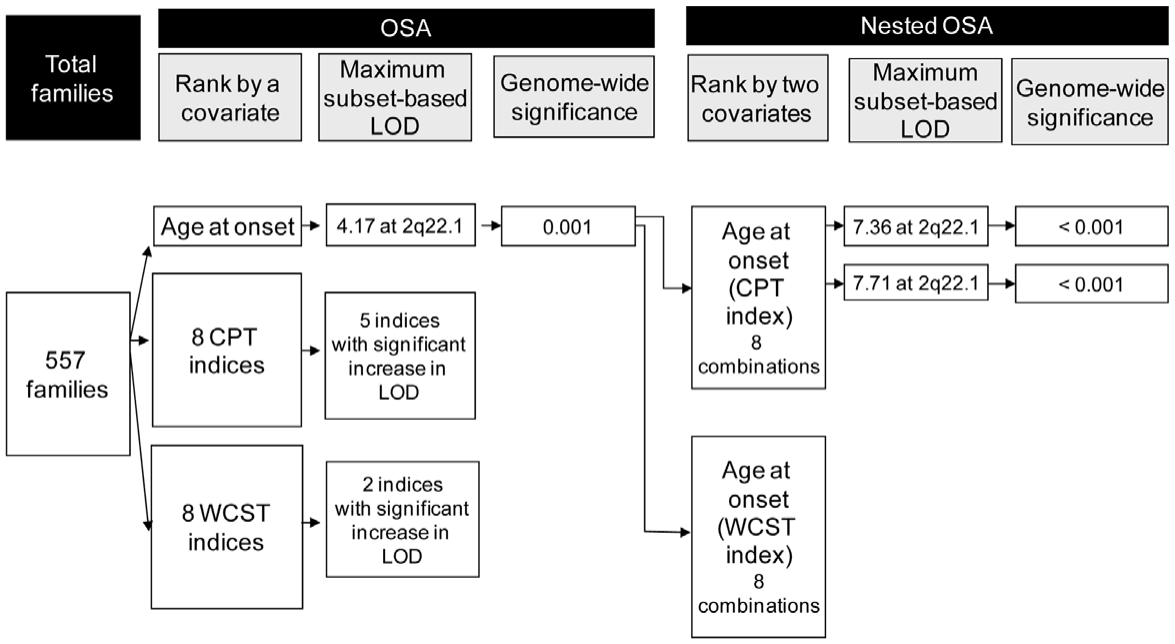
Flow chart of the ordered subset analysis (OSA) and nested OSA of genetic linkage in 557 families of siblings co-affected with schizophrenia. CPT, Continuous Performance Test; WCST, Wisconsin Card Sorting Test; LOD, logarithm of odds.

In this study, a series of family's covariates was explored, including the average values of the age at onset, CPT scores, and WCST scores from affected siblings. The whole procedures are depicted in the upper part of **Figure 1**. Of the original 557 families, 556 families had information on age at onset, 509 families on undegraded CPT scores, 499 families on degraded CPT scores, and 444 families on WCST scores, respectively. At the beginning of OSA, all the families were ranked according to the magnitude of a chosen family covariate, with those having the same covariate given the same rank. The genome-wide linkage analysis was then conducted in a series of subsets incrementally, starting with the rank that had the greatest family covariate. The procedure was repeated until all families were included in the final subset.

Since earlier age at onset as well as greater CPT-related or WCST-related deficits were associated with greater familial loading for schizophrenia, the following indices were ranked using ascending order: age at onset, four CPT indices (i.e., hit rate and d′ on both undegraded and degraded task), and three WCST indices (i.e., Categories Achieved, Conceptual Level Response, and Failure to Maintain Set). Meanwhile, the remaining indices were ranked using the descending order: four CPT indices (i.e., false alarm rate and reaction time on both undegraded and degraded task) and five WCST indices (i.e., Total Errors, Non-perseverative Errors, Perseverative Errors, Perseverative Responses, Trials to Complete First Category).

#### Chromosome-wide significance

**Figure 2:**
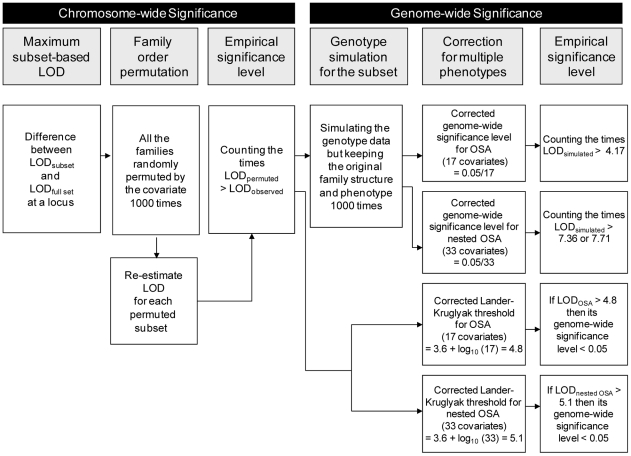
Flow chart of the estimation of chromosome-wide significance (left panel) and genome-wide significance (right panel) for a maximum subset-based logarithm of odds (LOD) in the ordered subset analysis (OSA) and nested OSA of genetic linkage in 557 families of siblings co-affected with schizophrenia.

The statistical significance of the difference between the greatest LOD scores in a subset of families identified by the OSA and the LOD scores at the same position in the full set of families was evaluated via 1,000 permutations. Under the null hypothesis that the ranking of the covariate is independent of the family's LOD scores on the target chromosome, the families were randomly permuted with respect to the covariate ranking and a chromosome-wide p value for each chromosome was yielded. A *P* value of ≤ 0.05 was considered significant, suggesting that a particular subset of individuals in the sample is more strongly linked to the specified chromosomal region than the whole sample. The procedures are summarized in the left part of **Figure 2**.

#### Nested ordered subset linkage analysis

Because OSA-derived subsets of families might have tied covariates, we attempted to introduce another subphenotype to further dissect the families (right part of **Figure 1**). In an OSA-derived subset of families characterized by a covariate that resulted in the greatest LOD scores, the families were further re-ranked by means of another subphenotype. The nested OSA procedure was repeated to seek a nested subset of families that resulted in a further increase in the linkage signal on the particular chromosome than the original subset. A *P* value of <0.05 in permutation was considered significant.

#### Genome-wide significance

After the permutation on the composition of a subset of families, which provides chromosome-wide significance level, we further evaluated its genome-wide significance by simulating the genotype data of the families and then estimating the empirical significance level with correction for multiple phenotypes, as illustrated in the right part of **Figure 2**. First, the genome-wide significance level of a subset-based maximum LOD score was derived from 1,000 simulations in our genotype data generated by the gene-dropping algorithm in Merlin. Simulated data were based on our original family structure, marker informativeness, spacing, and missing status, with phenotypic measurement and affection status being preserved. An *ad hoc* correction procedure was performed to account for the multiple informative phenotypes used in this study. For the OSA part, there were 17 covariates examined (including age at onset and 16 indices on the CPT and the WCST), and thus the threshold of a corrected genome-wide significance level was set as 0.0029 (i.e., 0.05/17). For the nested OSA part, there were 33 covariates examined (17 covariates in the OSA and 16 indices on the CPT and the WCST), and the threshold of a corrected genome-wide significance level was set as 0.0015 (i.e., 0.05/33).

Second, according to the Lander and Kruglyak threshold [32], which was also derived from simulations, a LOD score of greater than 3.6 indicates attaining genome-wide significance. To maintain the overall genome-wide significance level after considering multiple informative phenotypes used in this study, an *ad hoc* correction procedure was also performed, which was calculated as LOD_(corrected)_  =  3.6 + log_10_ (numbers of tests) [33]. Accordingly, the threshold of a LOD score reaching genome-wide significance was raised to 4.8 for the OSA part, and to 5.1 for the nested OSA part.

#### Family covariates comparison

The family covariate values between the subset of families that resulted in the greatest linkage signals and the remaining families were then compared. A linear mixed effect model with family treated as a random effect was applied using the Proc MIXED of the SAS software version 9.1 (SAS Institute, Cary, NC, USA) to adjust for within-family correlation.

## Results

The distributions of individual covariates, which were averaged over the affected siblings of each family, are listed in **Table 1**. On the basis of the adjusted z scores, the affected siblings had poorer performance across all of the indices that were based on the correctness of the response on both the CPT and WCST in comparison with the normative data. Meanwhile, the affected siblings had longer reaction time on both the undegraded and degraded CPT than the community norm. Furthermore, the measures of age at onset and neurocognitive performances employed in this study demonstrated significant familial resemblance, with an affected sib-pair correlation of 0.29 for age at onset (*P*<0.0001), 0.14 to 0.31 for CPT indices (*P* ranging from 0.004 to 0.0001), and 0.12 to 0.24 for WCST indices (*P* ranging from 0.04 to 0.0001).

**Table 1:** Distribution of age at onset, CPT scores, and WCST scores in affected siblings.

	Raw scores			Adjusted z scores^a^		
Variables	Mean (SD)	Q1^b^	Q3^c^	Mean (SD)	Q1^b^	Q3^c^
Age at onset (N = 1,085)	22.6 (6.3)	18.0	26.0	---	---	---
CPT-‘1-9’ indices						
Undegraded (N = 917)						
Hit rate	0.7 (0.3)	0.5	0.9	−1.9 (2.0)*	−3.9	−0.2
False alarm rate	0.1 (0.1)	0.0	0.1	1.5 (2.0)*	3.0	−0.1
d́	2.6 (1.5)	1.6	3.7	−2.3 (1.8)*	−3.9	−0.9
Reaction time (centi-sec)	45.1 (11.2)	37.0	51.0	1.0 (1.4)*	0.1	1.8
Degraded (N = 881)						
Hit rate	0.4 (0.4)	0.0	0.8	−2.5 (1.8)*	−4.2	−0.8
False alarm rate	0.1 (0.1)	0.0	0.1	1.2 (1.8)*	−0.2	2.2
d́	1.5 (1.6)	0.0	2.8	−2.6 (1.5)*	−3.9	−1.4
Reaction time (centi-sec)	53.6 (12.8)	45.0	66.0	1.0 (1.6)*	0.0	2.5
WCST indices (N = 718)						
Total Errors	73.4 (24.3)	55.0	95.0	1.4 (1.2)*	0.6	2.3
Non-perseverative Errors	29.2 (25.2)	11.0	38.0	0.5 (1.9)*	−1.0	1.2
Perseverative Errors	44.3 (28.4)	19.0	72.0	1.4 (1.8)*	−0.2	3.1
Perseverative Response	54.7 (39.3)	20.0	93.0	1.4 (1.9)*	−0.3	3.2
Categories Achieved	1.9 (2.5)	0.0	3.0	−1.0 (0.8)*	−1.6	−0.7
Conceptual Level Response	26.1 (24.1)	4.7	43.0	−1.3 (1.1)*	−2.2	−0.7
Failure to Maintain Set	0.8 (1.3)	0.0	1.0	−0.5 (1.0)*	−1.1	−0.2
Trials to Complete First Category	74.3 (54.2)	13.0	129.0	1.0 (1.5)*	−0.6	2.4

a The adjusted z scores were derived by means of standardizing the raw scores with adjustments for sex, age, and education against our norm data.

b Cut-off at the lowest 25% of data.

c Cut-off at the lowest 75% of data.

*P<0.0001 for the t-test examining if the adjusted z scores significantly different than 0.

**Table 2** summarizes the results of OSA that had a significant increase (empirical *P* ≤ 0.05) in a subset-based maximum LOD score from the initial LOD scores of the whole sample. The results of the OSA identified distinct chromosomal regions implicated for schizophrenia with different informative phenotypes: 1) 2q22.1 for younger age at onset; 2) 7q32.2, 8q13.1, and 9q34 for greater undegraded CPT-related deficits; 3) 7q32.3 for greater degraded CPT-related deficits; and 4) 8q13.1 and 8q21.1 for greater WCST-related deficits. For the five loci with significant increases in subset-based LOD scores, the percentage of linked families in each subset ranged from 10% to 53% of the whole sample. When the families within the subset were compared with the remaining families for the corresponding subphenotype, there were indeed significant differences between the two groups in the covariates, with earlier age at onset or more neurocognitive deficits in the subset of families. Of those covariate/locus combinations with a significantly increased LOD score, only “age at onset/2q22.1” reached genome-wide significance based on simulations after an *ad hoc* correction for multiple informative phenotypes, whereas no locus reached the Lander and Kruglyak genome-wide significance threshold after an *ad hoc* correction for multiple informative phenotypes.

**Table 2:** Ordered-subset analyses for schizophrenia by age at onset, CPT, or WCST (only results with a significant change in LOD scores are shown here).

			Change in LOD		Maximum subset-based		Mean family Covariates value (SE)	
Covariates	Cytogenetic location	Markers	(permutation p value)^a^	Initial LOD	LOD (genome-wide empirical p value) b	No. Families used/total (%)^c^	Within subset	Remaining families
Age at onset	2q22.1	D2S442	3.3 (0.007)	0.87	**4.17 (0.001)**	295/556 (53.1)	18.70 (0.16)**	26.86 (0.26)
CPT-‘1-9’ indices								
*Undegraded*								
Hit rate	8q13.1	D8S1136	2.25 (0.02)	0.95	3.20 (0.01)	160/509 (31.4)	−4.06 (0.08)**	−0.97 (0.06)
False alarm rate	7q32.3	D7S1804	1.80 (0.01)	1.25	3.05 (0.01)	95/509 (18.7)	1.87 (0.13)*	1.37 (0.07)
d′	8q13.1	D8S1136	0.95 (0.05)	1.83	2.78 (0.02)	158/509 (31.0)	−4.20 (0.06)**	−1.54 (0.07)
Reaction time	9q34	D9S2157	0.43 (0.01)	3.00	3.43 (0.02)	49/509 (9.6)	3.46 (1.00)**	0.81 (1.18)
*Degraded*								
Reaction time	7q32.3	D7S1804	1.35 (0.004)	2.30	3.65 (0.01)	161/499 (32.3)	2.46 (0.86)**	0.34 (1.39)
WCST indices								
Non-perseverative Errors	8q21.1	D8S1119	3.31 (0.004)	0.01	3.32 (0.01)	86/444 (19.4)	3.33 (0.13)**	−0.15 (0.06)
Categories Achieved	8q13.1	D8S1136	0.51 (0.05)	1.79	2.30 (0.07)	151/444 (34.0)	−1.63 (0.01)**	−0.70 (0.04)

a Families were randomly permuted for 1000 times with respect to the covariate ranking and a chromosome-wide p value for each chromosome was yielded.

b Significance level derived from simulations; a genome-wide empirical *P*-value <0.0029 [i.e., 0.05/(17 covariates)] is denoted in boldface as reaching genome-wide significance.

c The number of total families varied due to missing information on the covariate; subsets consisting of ≥ 15% of total families were reported here.

*P<0.001, **P<0.0001, for the mixed effect model comparing the families covariate values between the subset of families and the remaining one.

Among the loci identified in **Table 2**, the greatest LOD scores of 4.17 at D2S442 (147 cM) was found in 53% of the families (n  =  295, mean age at onset  =  18.70) ranked by age at onset. Since many of the families had the same mean values of age at onset, we were interested in whether a further subsetting by one CPT or WCST index could result in further increase in the LOD score on the same chromosomal region. Before the subsetting, we examined the relations of age at onset to either a CPT or WCST index and found no significant correlations in any of them (data not shown). As displayed in **Table 3**, among these CPT-related and WCST-related deficits, only undegraded and degraded CPT false alarm rate reached significant increase in the nested OSA-based LOD on 2q22.1, with a score of 7.36 (empirical *P* = 0.004) in 81% of 282 families (mean adjusted z scores of false alarm rate  =  1.84, mean age at onset  = 18.40) and 7.71 (empirical *P* = 0.001) in 87% of 281 families (mean adjusted z scores of false alarm rate  =  1.49, mean age at onset  = 18.40), respectively. Of note, both the LOD score of 7.36 (nominal *P*<0.00001) and of 7.71 (nominal *P*<0.00001) reached genome-wide significance, regardless of which method was used in the significance evaluation, i.e., simulated genotype data or the adjusted Lander and Kruglyak threshold after accounting for multiple phenotypes. More severe CPT or WCST deficits were indeed found in the families of the nested subset than those in the remaining families, as summarized in **Figure 1**.

**Table 3:** Nested ordered-subset analysis in the subgroup with early-onset schizophrenia by CPT or WCST.

			Change in LOD		Maximum subset-based		Mean family covariate value (SE)	
Covariates	Cytogenetic location	Markers	(permutation p value)^a^	Initial LOD	LOD (genome-wide empirical p value)^b^	No. Families used/total (%)	Within nested subset	Remaining families
CPT-‘1-9’ indices								
Undegraded hit rate	2q22.1	D2S442	0.23 (0.54)	4.23	4.46	275/282 (97.5)	---	---
Undegraded false alarm rate	2q22.1	D2S442	3.13 (0.004)	4.23	**7.36 (<0.001)**	228/282 (80.9)	1.84 (0.09)*	-0.04 (0.03)
Undegraded d′	2q22.1	D2S442	0.85 (0.08)	4.23	5.08	206/282 (73.0)	---	---
Undegraded reaction time	2q22.1	D2S442	0.48 (0.38)	4.23	4.71	268/282 (95.0)	---	---
Degraded hit rate	2q22.1	D2S442	0.70 (0.16)	4.23	4.93	242/281 (86.1)	---	---
Degraded false alarm rate	2q22.1	D2S442	3.48 (0.001)	4.23	**7.71 (<0.001)**	243/281 (86.5)	1.49 (0.09)*	-0.26 (0.04)
Degraded d′	2q22.1	D2S442	1.06 (0.09)	4.23	5.29	251/281 (89.3)	---	---
Degraded reaction time	2q22.1	D2S442	1.13 (0.07)	4.23	5.36	262/281 (93.2)	---	---
WCST indices								
Total Errors	2q14.1	D2S410	0.53 (0.08)	3.84	4.37	67/263 (25.5)	---	---
Non-perseverative Errors	2q22.1	D2S442	0.68 (0.16)	3.64	4.32	247/263 (93.9)	---	---
Perseverative Errors	2q22.1	D2S442	0.00 (1.00)	3.64	4.17	263/263 (100.0)	---	---
Perseverative Response	2q22.1	D2S442	0.00 (1.00)	3.84	3.84	263/263 (100.0)	---	---
Categories Achieved	2q14.1	D2S410	0.56 (0.34)	3.18	3.74	55/263 (20.9)	---	---
Conceptual Level Response	2q14.1	D2S410	0.41 (0.40)	3.18	3.59	72/263 (27.4)	---	---
Failure to Maintain Set	2q22.1	D2S442	0.69 (0.47)	3.64	3.87	255/263 (97.0)	---	---
Trials to Complete First Category	2q22.1	D2S442	0.76 (0.15)	3.64	4.40	53/263 (20.2)	---	---

a Families were randomly permuted for 1000 times with respect to the covariate ranking and a chromosome-wide p value for each chromosome was yielded.

b Significance level derived from simulations; a genome-wide empirical p-value <0.0015 [i.e., 0.05/33 covariates)] is denoted in boldface as reaching genome-wide significance.

*P<0.0001 for the mixed effect model comparing the families covariate values between the nested subset of families and the remaining ones.

**Figure 3:**
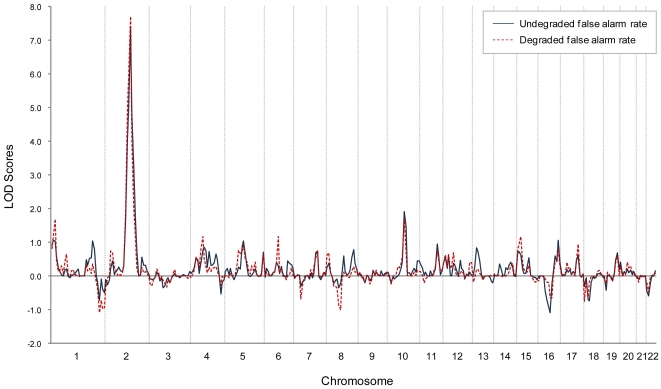
Genome-wide nonparametric LOD scores of two indices attaining genome-wide significance in the nested OSA linkage analyses over 22 autosomal chromosomes.

**Figure 4:**
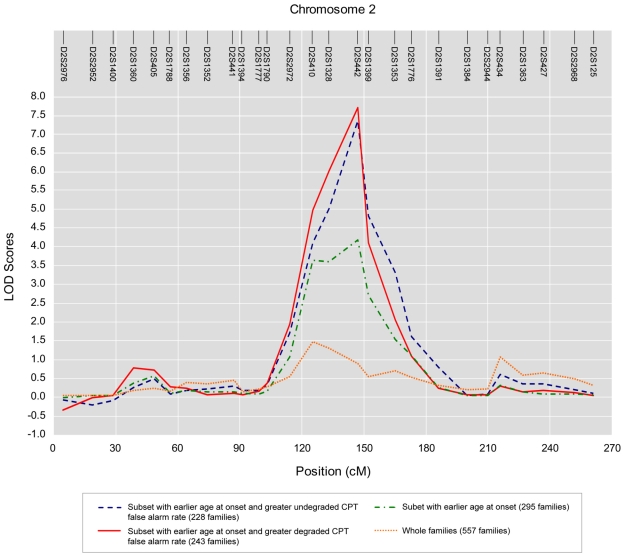
The result of OSA linkage analysis in the subset of families defined by a variety of covariates, including earlier age at onset, greater undegraded CPT false alarm rate nested within earlier age at onset, greater degraded CPT false alarm rate nested within earlier age at onset, versus that of the original linkage analysis of the whole sample on chromosome 2.

The distributions of the genome-wide LOD scores of the nested OSAs that were based on age at onset and CPT false alarm rates over 22 autosomal chromosomes are illustrated in **Figure 3**. The corresponding chromosome-wide LOD distributions on chromosome 2 from the results of OSA and nested OSA are shown in **Figure 4**. Compared with the initial linkage analysis for schizophrenia using all available families, OSA using age at earlier onset as covariate did strengthen and narrow the linkage peak. Moreover, the strengthening pattern was further augmented when additionally incorporating undegraded or degrade CPT false alarm rate as another family covariate in the nested OSA.

## Discussion

This study found that subsets of families of patients with schizophrenia characterized by younger age at onset or neurocognitive deficits exhibited increased linkage signals and reached genome-wide significance on 2q22.1, which was nearby a suggestive locus (a LOD score of 1.16 on 2q14.1) in the initial genome-wide scan for the whole sample [21]. Furthermore, a subset with greater CPT deficit nested within the families with younger age at onset increased the linkage signal on 2q22.1 to a LOD score as high as 7.71. The results have provided intriguing clues for gene search for a complex disease as schizophrenia.

Our nested OSA approach demonstrates an exploratory way to incorporate various schizophrenia-related characteristics in deciphering the genetic linkage signal to schizophrenia for families with tied covariates (i.e., the same mean values of age at onset). Our results revealed that in the onset-based subset of families having maximal linkage signals, families with tied covariates might in fact have linkage scores in the opposite directions. Accordingly, a further subsetting by one CPT or WCST index might partition the families into more homogeneous subsets and then result in more increase in the LOD scores. Unlike other statistical methods for detecting linkage by dissecting heterogeneity, which require a prior stratification of the data [34] and do not consider quantitative trait information [35], the OSA approach takes advantage of disease-related phenotype information and has been successfully applied in other complex diseases [36], [37].

An important feature of this study is that we used age at onset and several facets of the CPT and WCST as covariates to reduce phenotypic and genetic heterogeneity of schizophrenia. These are important features as they clearly impact the outcome and severity of the illness. In addition, cognitive deficit is a core symptom for schizophrenia [38] and the endophenotypic nature of this deficit, more so for CPT [10] than for WCST performance [29], may help index underlying genetic liability or vulnerability of the disease. Particularly, greatest increases in LOD scores were obtained for the families of schizophrenia patients with more CPT false alarm rates nested within the families with younger age at onset. Our findings were consistent with previous studies indicating that some genetic variants were associated with both younger age at onset and more severe cognitive deficits of schizophrenia [39]. Therefore, the combination of clinical and cognitive features used in this study may aid the identification of susceptibility and modifying genes for schizophrenia.

Unlike previous linkage studies for psychiatric disorders that used selective indices of neurocognitive tasks, either as a composite profile of neurocognitive performance [40], [41] or part of multivariate traits [42], this study used all available indices that exhibited significant familial resemblance. Our approach might avoid missing potentially important endophenotypes, such as CPT reaction time [43] or CPT hit rate [44]. Although the psychometric construction of the indices on the tests might be inter-correlated, the cognitive deficits underlying individual indices might be attributed to quite different psychopathologies. For example, previous studies indicated that individual CPT or WCST indices are differentially associated with different symptom dimensions for schizophrenia [43], [45], [46] or schizotypy [26], [47]. In addition, the brain dysfunctions underlying WCST perseverative errors might be different from those underlying nonperseverative errors [9], [48], [49], [50].

However, our approach might suffer from the issue of multiple testing. To fully address this, several strategies were used in this study to help interpret the significance of the OSA results. First, we used permutation tests to estimate the statistical significance of the change in LOD score from the subsets, which controlled for inflation in the false-positive rate induced by examining multiple family subsets for a given covariate. Second, two simulation-based strategies were applied to estimate the genome-wide significance level of subset-based LOD scores, with one based on the simulated genotype data of this sample and the other one based on adjusting the Lander-Kruglyak threshold that was also obtained by simulations. Such simulations can help control for the inflation in the false-positive rate over multiple chromosomes and markers since genome-wide significance was estimated by the proportion of times that a particular LOD score was reached anywhere in the simulated genome. Finally, we used a Bonferroni correction to adjust for multiple trait-related covariates after empirical genome-wide *P* values were derived from simulations. The current results of particular subset-based NPL-Z scores attaining genome-wide significance might be very conservative because a Bonferroni-type correction of the *P* value was applied for the correlated indices among the CPT or WCST. It should be pointed out that it is very difficult to assess the statistical significance of our subset-based linkage signals due to the complexity inherent in the many possible sources of multiple testing. Accordingly, the results reported here might be better treated as exploratory and independent replication is warranted to reassure the robustness of the findings.

The most striking linkage signal found in this study was obtained from a nested OSA on chromosome 2q22.1 (D2S4422 at 147.4 cM). Intriguingly, a rank-based meta-analysis showed evidence of linkage in this region [51]. A recent genome-wide association study also found several SNPs on 2q22.1 to be associated with schizophrenia [52]. What this study adds is that this locus was likely to be involved with not only younger age at onset but also greater CPT deficit in false alarm rate in schizophrenia. The region contains potential susceptibility genes for schizophrenia, like HNMT (Histamine N-methyltransferase) and NR4A2 (alias NURR1, nuclear receptor subfamily 4, group A, member 2, an orphan nuclear receptor and putative transcription factor for the dopamine transporter). Although previous studies found that neither HNMT [53] nor NR4A 2 [54], [55], [56] were associated with schizophrenia, the negative findings might be attributed to the lack of sub-grouping by particular quantitative traits in these studies. Further fine-mapping studies using SNP arrays or deep resequencing methods among patients with early age at onset and CPT deficits may help identify the common genetic variants or rare coding sequence variants that are involved in the genetic susceptibility to this subtype of schizophrenia.

This study is limited in several aspects. First, our samples consisted of adults with Han Chinese ancestry only, and hence the results may not extrapolate to populations of other ethnicities where allele frequencies may vary. Second, despite our use of different strategies to deal with multiple testing, our linkage findings may not rule out the possibilities of false positive or false negative because genome-wide statistical significance of OSA-derived subset results remains a difficult issue. Third, the age at onset of schizophrenia in this study was determined mainly on the initiation of first psychotic symptoms. Although the age at first functional impairment may be closer to the true onset of the disease, there is an overall agreement that the onset of the first psychotic episode is a better marker for the onset of schizophrenia because it has a higher specificity than negative or nonspecific symptoms [57].

In summary, we obtained possible evidence of linkage on chromosome 2q22.1 in families of schizophrenia patients with more CPT false alarm rates nested within the families with younger age at onset. Our results suggest the presence of etiologic heterogeneity in schizophrenia and highlight the importance of unraveling the complex genetics of schizophrenia by incorporating genetically informative phenotypes.
